# Impaired Cognitive Empathy in Outpatients with Chronic Musculoskeletal Pain: A Cross-Sectional Study

**DOI:** 10.1155/2021/4430594

**Published:** 2021-09-25

**Authors:** Hang-Bin Zhang, Hang Ou, Dian-Huai Meng, Qian Lu, Lei Zhang, Xi Lu, Zhi-Fei Yin, Chuan He, Ying Shen

**Affiliations:** ^1^Department of Rehabilitation Medicine, The Affiliated Jiangsu Shengze Hospital of Nanjing Medical University, Suzhou, Jiangsu, China; ^2^Shanghai Key Laboratory of Psychotic Disorders, Shanghai Mental Health Center, Shanghai Jiao Tong University School of Medicine, Shanghai, China; ^3^Research Center of Brain and Cognitive Neuroscience, Liaoning Normal University, Dalian, Liaoning, China; ^4^Key Laboratory of Brain and Cognitive Neuroscience, Liaoning, China; ^5^Rehabilitation Medicine Center, The First Affiliated Hospital of Nanjing Medical University, Nanjing, Jiangsu, China; ^6^Department of Medical Psychology, School of Mental Health and Psychological Sciences, Anhui Medical University, Hefei, Anhui, China; ^7^Department of Rehabilitation Medicine, China-Japan Friendship Hospital, Beijing, China

## Abstract

**Background:**

In recent years, a growing number of researchers showed significant interest in psychological and social interventions to manage chronic musculoskeletal (MSK) pain. Cognitive and emotional empathy is an attractive and valuable sociopsychological factor that may provide protection and resilience against chronic MSK pain. However, its effect on outpatients remains underexplored.

**Objective:**

To compare the empathy ability between chronic MSK pain outpatients and healthy controls and explore the relationship between cognitive/emotional empathy and chronic pain.

**Methods:**

Patients with chronic MSK pain (*n* = 22) and healthy controls (*n* = 26) completed the pain assessment and empathy ability task, utilizing a multidimensional empathy assessment tool with satisfactory reliability and validity (i.e., the Chinese version of the Multifaceted Empathy Test (MET-C)).

**Results:**

The data indicated that the chronic MSK pain outpatients had impaired cognitive empathy (i.e., lower squared cognitive empathy accuracy: Student's *t* = −2.119, *P* = 0.040, and longer task completion time: Student's *t* = 3.382, *P* = 0.002) compared to healthy controls, and cognitive empathy was negatively correlated with pain intensity (*r* = −0.614, *P* = 0.002). Further, the impaired cognitive empathy was present in identifying positive, but not negative emotions.

**Conclusion:**

These results indicate that chronic MSK pain is associated with impaired empathy ability. Our studies contribute to offering a potential direction for developing psychosocial interventions to treat chronic MSK pain.

## 1. Introduction

Chronic musculoskeletal (MSK) pain is the main contributor to disability worldwide [1]. According to the World Health Organization (WHO), 20–33% of the world's population (1.75 billion people) has some form of chronic MSK pain [1]. Chronic MSK pain was commonly defined as pain persisting for longer than 3 months, and it may be due to sustained stimulation of nociceptors damaged in areas of persistent tissue damage (i.e., bones, muscles, ligaments, tendons, and even nerves) [2]. Chronic MSK pain results in great suffering among patients and poses an immense global socioeconomic burden [3]. Although it increases suffering in daily activities, drug consumption, and high frequency of sick leave and disability pensions, there is no consensus on the mechanism underlying chronic MSK pain, and the current targeted medical treatments have limited efficacy; therefore, further research on chronic MSK pain is required [4].

Most MSK patients have to live with pain for a long duration, and the continuous physical suffering and social stigmatization from MSK pain decrease their quality of life [5]. Chronic MSK pain is also often accompanied by mental health problems, such as depression, anxiety, emotional regulation problems, and sleep disorders, as well as impaired cognitive function (e.g., decreased inhibitory control, memory and, in particular, emotion-related ability), which might impair social function [6, 7]. This social dysfunction and the accumulating chronic pain itself would alter neural circuits involved in cognitive and emotional control, exacerbating the chronic pain or causing a transition to severe neuropathic pain [8, 9]. In this context, the biopsychosocial model, which posits that chronic pain is a multidimensional disorder that involves the interaction of physiological, psychological, and social factors, is the most widely acceptable and reliable theory for chronic pain [10, 11]. In this framework, chronic MSK pain is deemed to be associated with psychological and social processes which, in turn, greatly impact the feeling of pain in muscles and the skeleton [12].

One of the most valuable and attractive indicators of social function is empathy, which is pivotal to social relationships and is an important factor that influences the quality of life [13]. Thus, besides the use of pharmacological interventions (either as monotherapies or combination therapies), empathy ability, which may be a protective factor related to the psychological and social aspects of chronic pain, needs to be assessed and analyzed [14]. Empathy comprises cognitive empathy (mental perspective-taking: emotion recognition and theory of mind) and emotional empathy (vicarious sharing of emotion: affective sharing) [15]. Empathy ability predicted self-perceived social support and positive life changes, which allows resilience in response to chronic pain [16]. Additionally, a study reported that patients with chronic low back pain had impaired empathy (as measured by the Basic Empathy Scale in Adults) [17], and improving empathy ability improves interpersonal relationships and quality of life [18].

However, little empirical evidence demonstrates links between empathy ability and specific dimensions of chronic pain (such as pain intensity and duration) [19], especially for outpatients. In addition, these studies usually assessed empathy using single-dimension questionnaires, so they failed to accurately determine the level of empathy among chronic MSK pain patients [20]. Accordingly, the Multifaceted Empathy Test (MET), which provides a more stable estimation of empathy and involves photorealistic stimuli, is recommended [21]. The use of the MET may deepen clinicians' understanding of patients' cognitive empathy and emotional empathy, contributing to both research and clinical decision making.

We recruited outpatients with chronic MSK pain, which is a major type of chronic pain, in this study. The principal objective was to investigate the multidimensional empathy ability (assessed using the self-reported Interpersonal Reactivity Index (IRI) and the MET-C) of these chronic MSK pain outpatients compared to healthy controls (HCs) and the relationships between chronic MSK pain and pain-related factors (pain duration, pain intensity, sleep quality, and emotion alterations).

## 2. Materials and Methods

### 2.1. Participants

**Figure 1:**
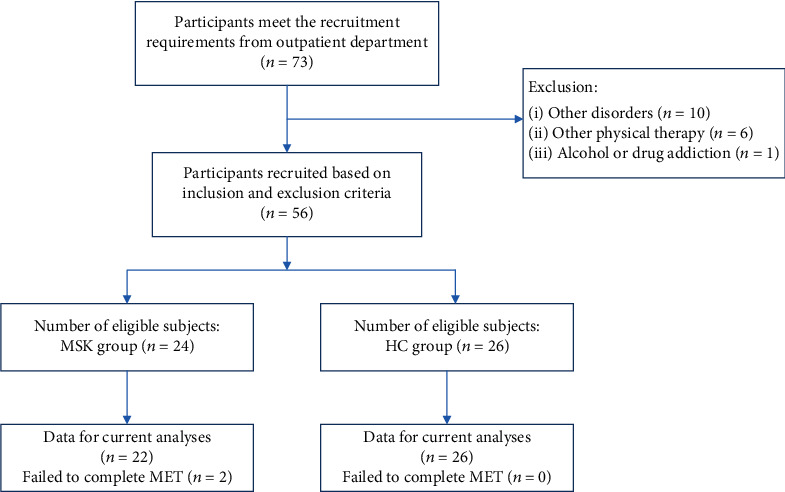
The flow chart of the patients.

Twenty-two outpatients with chronic MSK pain (5 males and 17 females; mean age ± SD, 44.41 ± 7.94 years) and twenty-six healthy people (HCs; 8 males and 18 females, mean age ± SD, 40.08 ± 10.86 years) with the same gender distribution and age range participated in the study. In a brief patient consultation, all detailed information related to each patient's pain was recorded. The criteria for inclusion were (i) with primary chronic MSK pain according to the International Association for the Study of Pain Classification of Chronic Pain for the International Classification of Diseases [22], including the pain in the shoulder, leg, arm, and back; (ii) aged 18-60; (iii) course of disease ≥ 3 months with pain intensity > 3/10 Numerical Rating Scale (NRS); and (iv) provision of informed consent. The exclusion criteria were as follows: (i) other major physical or mental disorders or other types of chronic pain (including neuralgia or visceral pain); (ii) alcohol or drug addiction; (iii) participated in other physical therapy within the past 3 months; (iv) recently received major surgical treatment; and (v) enrolled in any other rehabilitation program. The flow chart of patients is shown in Figure 1.

All participants voluntarily signed informed consent forms prior to recruitment and were compensated $15 after completing all the questionnaires. The study was approved by the Ethics Committee of the Affiliated Jiangsu Shengze Hospital of Nanjing Medical University (JSSZYY-LLSC-202019) and was registered with the China Clinical Trial Registration Center (http://www.chictr.org.cn) under the number ChiCTR2000041062.

### 2.2. Questionnaires

The participants filled out standardized questionnaires. The questionnaires quantitatively assessed pain and empathy (as described below). To eliminate the effect of interference factors which are common and specific in chronic pain patients, the emotions, sleep quality, and mental state of subjects in both groups were also evaluated using the Positive and Negative Affectivity Scale (PANAS), Pittsburgh Sleep Quality Index (PSQI), and Depression, Anxiety, and Stress Scales–21 Items (DASS-21), respectively [23, 24]. The anatomical pain sites of the patients are presented in Table 1.

### 2.3. Pain Assessment

We assessed both pain duration and pain intensity, which was evaluated using two pain scales: the 11-point NRS and the Short-Form McGill Pain Questionnaire (SF-MPQ). The NRS is used worldwide as a valid measure of pain intensity with promising clinical value for chronic pain patients [25]; the SF-MPQ allows comprehensive assessment of pain quality (based on sensory and affective dimensions of pain experience) and intensity [26]. The SF-MPQ comprises a list of pain adjectives and is considered a more reliable and valid index of an individual's pain experience than other self-reported measures [27]. Multidimensional assessments of pain can reduce potential errors associated with assessment tools.

### 2.4. Empathy Ability

**Figure 2:**
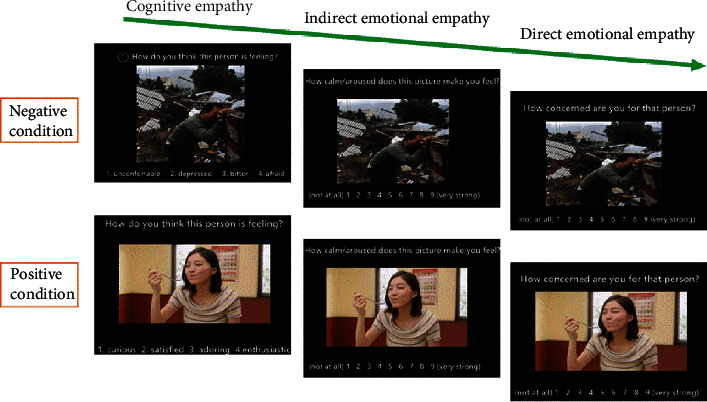
Example items of the MET-C.

First, participants' trait cognitive empathy and trait emotional empathy were measured using the IRI, which has four subscales: the perspective-taking and fantasy subscales represent cognitive empathy, while the empathic concern and personal distress subscales represent emotional empathy [28, 29]. Second, as the MET has higher ecological validity for assessing cognitive empathy and emotional empathy than self-reported questionnaires [30], the Chinese version of this task (MET-C) was also used. It involves 40 pictures of people in various emotional states (20 positive and 20 negative emotional valence pictures). After seeing each picture, participants were asked to respond to three questions. First, to assess cognitive empathy accuracy and task completion time, for each picture, they were presented with four words describing four emotions and were asked to select the one that best fits the picture. Next, to assess emotional empathy, participants were asked “How calm/aroused does this picture make you feel?” (indirect emotional empathy) and “How concerned are you for this person?” (direct emotional empathy) on a scale of 0 (not at all) to 9 (very much). The procedures followed those set out by Wu et al. [30] (Figure 2).

### 2.5. Statistical Analyses

Data were analyzed using STATA software version 15.1 (Stata Corporation, USA). The chronic MSK pain and HC groups were compared using the independent-samples *t*-test or Pearson's chi-square test, as appropriate. To assess the between-group differences in empathy in the positive or negative emotional valence conditions, repeated-measures analysis of variance (ANOVA), followed by post hoc Bonferroni tests, was used. Pearson correlation analyses were also used to assess associations between empathy (i.e., cognitive empathy accuracy, based on the MET-C) and other variables (pain intensity (SF-MPQ), pain duration, positive/negative emotion (PANAS), sleep quality (PSQI), age, education level, or MET-C task completion time). Multivariate stepwise linear regression was used to assess whether pain factors (SF-MPQ and pain duration) and demographic factors (age and education level) can predict empathy (i.e., cognitive empathy accuracy, based on the MET-C). Data were inspected for normality using the Shapiro–Francia test. Two-tailed *P* value < 0.05 was considered significant.

## 3. Results

### 3.1. Demographic and Clinical Characteristics

The detailed demographic and clinical information of the participants is shown in Table 2. There were no significant differences between the chronic MSK pain and HC groups in age (*P* = 0.128), education level (*P* = 0.102), gender (*P* = 0.532), or handedness (*P* = 0.272), based on independent-samples *t*-tests and chi-square tests. Regarding clinical characteristics, the chronic MSK pain group had poorer sleep quality than the HC group (*P* = 0.014), while no significant difference was found in positive and negative emotion (positive: *P* = 0.351; negative: *P* = 0.058) or in depression (*P* = 0.122), anxiety (*P* = 0.087), and stress (*P* = 0.536). The Shapiro–Francia test showed that the mean cognitive empathy accuracy (HC group: *P* = 0.033; chronic MSK pain group: *P* = 0.005) and accuracy in positive (HC group: *P* = 0.084; chronic MSK pain group: *P* = 0.202) and negative (HC group: *P* = 0.400; chronic MSK pain group: *P* = 0.008) emotion conditions had nonnormal distributions, while the squared mean cognitive empathy accuracy (HC group: *P* = 0.258; chronic MSK pain group: *P* = 0.068) and squared mean accuracy in positive (HC group: *P* = 0.448; chronic MSK pain group: *P* = 0.678) and negative (HC group: *P* = 0.738; chronic MSK pain group: *P* = 0.153) emotion conditions had normal distributions. Hence, we used the squared value in the analysis.

### 3.2. Inconsistency between IRI and MET-C

**Figure 3:**
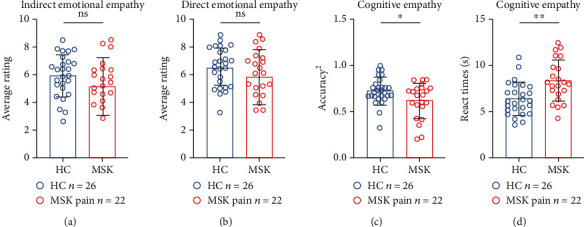
Comparisons of the indirect emotional empathy score (a), direct emotional empathy score (b), squared cognitive empathy accuracy (c), and task completion time for cognitive empathy section (d) between chronic musculoskeletal pain (MSK pain) and healthy control (HC) groups. ns: no significant; ^∗^*P* < 0.05; ^∗∗^*P* < 0.01.

**Figure 4:**
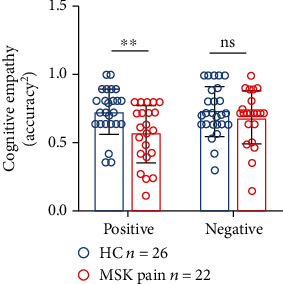
Comparisons of squared cognitive empathy accuracy between chronic musculoskeletal pain (MSK pain) and healthy control (HC) groups in positive/negative emotional valence conditions. In the positive condition, the squared cognitive empathy accuracy was significantly lower in the MSK pain group than the HC group, while there was no significant difference in the negative condition. ns: no significant; ^∗∗^*P* < 0.01.

There were no significant differences between the chronic MSK pain and HC groups in IRI trait empathy (disposition to empathic responsiveness according to a self-reported questionnaire; Table 3), comprising mean self-reported cognitive empathy (perspective-taking: Student's *t* = 0.442, *P* = 0.660; fantasy: Student's *t* = 0.282, *P* = 0.779) and mean self-reported emotional empathy (empathic concern: Student's *t* = −0.039, *P* = 0.969; personal distress: Student's *t* = −0.058, *P* = 0.954). Similarly, according to the MET-C, there were no significant differences in either the mean indirect emotional empathy (Figure 3(a); Student's *t* = −1.472, *P* = 0.148) or mean direct emotional empathy (Figure 3(b); Student's *t* = 1.345, *P* = 0.185). However, the MET-C revealed impaired cognitive empathy in chronic MSK pain patients compared to HCs: the chronic MSK pain group had a lower squared cognitive empathy accuracy (Figure 3(c); Student's *t* = −2.119, *P* = 0.040) and a longer task completion time (Figure 3(d); Student's *t* = 3.382, *P* = 0.002). The repeated-measures ANOVA indicated a significant main effect of group (*F* = 4.614, *P* = 0.037, *η*^2^ = 0.091), emotion valence (*F* = 5.660, *P* = 0.022, *η*^2^ = 0.110), and interaction term (*F* = 4.254, *P* = 0.045, *η*^2^ = 0.085). Post hoc analysis showed that the impaired cognitive empathy was pronounced in the positive emotion condition (*F* = 9.105, *P* = 0.004, *η*^2^ = 0.165) but there was no difference in cognitive empathy in the negative emotion condition (*F* = 0.055, *P* = 0.816, *η*^2^ = 0.001; Figure 4).

### 3.3. Correlation between Pain and Empathy

**Figure 5:**
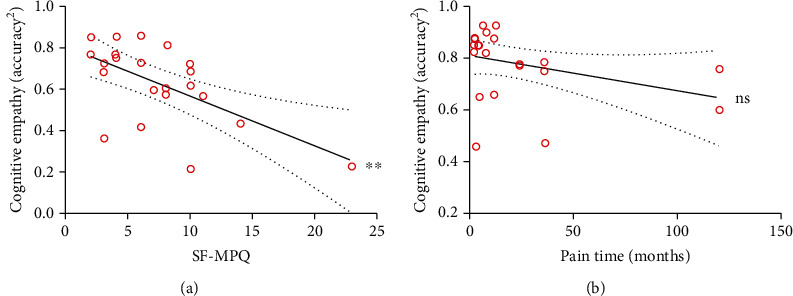
Correlations of SF-MPQ with squared cognitive empathy accuracy and pain duration. (a) Positive correlation between SF-MPQ and squared cognitive empathy accuracy. (b) No correlation between pain duration and cognitive empathy accuracy. The thick line indicates the regression line, and the dotted arcs indicate the confidence limits. ns: no significant; ^∗∗^*P* < 0.01.

Pearson correlation analysis showed a significant negative correlation between pain intensity (SF-MPQ) and squared cognitive empathy accuracy (MET-C; *r* = −0.606, *P* = 0.003), but not between pain duration and squared cognitive empathy accuracy (Table 4). Additionally, stepwise multivariate linear regression showed that only pain intensity (SF-MPQ) was significantly associated with squared cognitive empathy accuracy (adjusted *R*^2^ = 0.335, *P* = 0.003, *b* = −0.024; Figure 5(a)), but pain duration and other demographics were not (Figure 5(b)). There were also no correlations between squared cognitive empathy accuracy (either mean accuracy or accuracy in the positive or negative conditions) and positive/negative emotion (PANAS), sleep quality (PSQI), age, or education level (Table 4).

## 4. Discussion

This study revealed the impaired cognitive empathy (i.e., lower squared cognitive empathy accuracy and longer task completion time based on the MET-C) in chronic MSK pain patients compared to HCs. This is consistent with previous research on chronic MSK pain, which found impaired empathy (as measured by the Basic Empathy Scale in Adults) in patients with chronic low back pain [17]. However, we found that the chronic MSK pain patients' emotional empathy was not impaired. This inconsistency between cognitive empathy and emotional empathy in patients has also been reported in adults with Asperger's syndrome (one of the primary symptoms: impaired social interaction), indicating that these individuals with chronic MSK pain can also be confused by others' emotions [21]. Besides, impaired cognitive empathy with good emotional empathy was thought to be a psychotic symptom, which in chronic MSK pain can also result in interpersonal problems and social stigmatization for patients [31]. In summary, using a behavior task (i.e., the MET-C), we found that impaired cognitive empathy (without impaired emotional empathy) was evident in the chronic MSK pain patients, which may lead to a decline in prosocial behavior.

In addition, the finding regarding the impaired cognitive empathy in chronic MSK pain patients was also supported by the correlation analysis, which demonstrated that pain intensity (SF-MPQ) was negatively correlated with cognitive empathy accuracy, as in a previous study [18]. However, there was no correlation between pain duration and cognitive empathy accuracy. This means that even in the early stage of chronic MSK pain, cognitive empathy possibly has declined to a low level, and pain intensity rather than pain duration is associated with the degree of cognitive empathy impairment. This result highlights the importance of identifying impairments in empathy with a suitable assessment tool in the early stage of chronic pain. Compared to the subjective NRS, the SF-MPQ has more descriptive details about types of pain sensations (e.g., throbbing, shooting, stabbing, and fear), and it is a more objective multidimensional measure [32]. Based on the correlation between pain intensity (SF-MPQ) and empathy (cognitive empathy accuracy, based on the MET-C), it might indicate that the SF-MPQ provided additional information about the level of ability to recognize emotions.

Furthermore, the results revealed that cognitive empathy impairment was linked to emotional valence. The chronic MSK pain patients did not report an intensely subjective experience of emotion (i.e., PANAS) in either the positive or negative emotion conditions. Patients had the same cognitive empathy accuracy as HCs (i.e., they recognized/understood the emotions) in the negative emotion condition, and the chronic MSK pain and HC groups had the same direct and indirect emotional empathy in the negative emotion condition. In contrast, patients had significantly impaired cognitive empathy accuracy in the positive emotion condition compared to HCs, though these groups had the same direct and indirect emotional empathy in the positive emotion condition. This might be caused by compensation for the impaired empathetic recognition (i.e., impaired cognitive empathy accuracy) in the positive emotion condition [33]. From this, it can be seen that accurately detecting the levels of empathy ability and pain requires that appropriate comprehensive assessment methods are used.

Notably, there were no significant between-group differences in IRI, a typical self-reported scale for assessing cognitive empathy and emotional empathy, despite there being a significant difference according to the MET-C. Thus, by utilizing the MET-C, which involves visual stimulation encompassing different emotions, we highlighted the applicability of a more objective empathy assessment in chronic pain patients.

The PANAS scores and even the DASS-21 scores did not differ significantly between groups, which conflicts with previous studies demonstrating significant increases in depression and stress in chronic MSK pain patients [24, 34]. This may be readily explained by the findings of Cruz-Almeida et al. of large individual differences in pain and psychological function [35]. Analysis of chronic pain patient subgroups with specific sets of clinical characteristics is needed to fully explore differences within the chronic pain patient population. In addition, some of the previous research involved hospital patients with severe illness, with more psychological and somatic symptoms and poorer quality of life. The difference in clinical status and treatment settings may explain the inconsistencies with previous research [36]. Changes in a patient's normal living environment (including changes in social and affiliative behaviors) are not conducive to studying empathy or reflecting the actual patient situation. We recruited chronic MSK pain outpatients (who were not hospitalized and thus had similar social circumstances to the HCs) to make the comparison more reliable; thus, the differences observed in our data mostly reflect the presence of chronic MSK pain rather than other factors. Furthermore, another study suggested that chronic pain in different body regions might be reflective of different brain signatures [37], which may also help to explain the inconsistencies between our results (involving no significant differences in PANAS or DASS-21 between chronic MSK pain patients and HCs) and the previous results. As our findings highlight that, for chronic pain, early detection of impaired empathy and preventive strategies are particularly important, our findings on empathy may provide value for ambulatory chronic pain patients.

Our findings also concur with neurological research on chronic pain. Regarding general MSK chronic pain, the main dysfunctional cortices are the cingulate, prefrontal, and primary/secondary somatosensory cortices [38], and these impaired brain areas are also involved in empathic processing (e.g., the medial/lateral prefrontal cortex conceivably mediates empathy by processing information and action-relevant stimuli [39], and the primary somatosensory cortex plays an important role in both actual pain perception and social recognition [40]). These facts provide a hint about the connectedness between empathy and chronic MSK pain and a potential intervention target for impaired empathy in chronic MSK pain patients.

Previous research on the relationship between chronic pain and empathy has focused on the effect of observers' empathy for chronic pain patients rather than the empathy ability of patients themselves [41]. Various literatures on pain and empathy have demonstrated that the failure of surgery was more likely attributed to patient's psychological dysfunction [42]; the effect of intervening was correlated with the empathy they have perceived [43]. However, these models have focused on the empathy of observers rather than the patients', while trying to control relations with others to improve patient's symptoms seems unlikely. Considering the model of pain and empathy and the fact that patients' own empathy ability is highly positively correlated with their social support system [44], our results potentially provide a novel approach for the treatment of chronic MSK pain. That said, focusing on patients' empathy ability makes chronic MSK pain treatment through controlling social factors possible and feasible.

There are several limitations to our study. First, the results are limited due to the relatively small sample size and cross-sectional design. Although an association was identified, we cannot prove causality regarding the effect of empathy on chronic MSK pain without longitudinal follow-up. Second, a single behavior task (MET-C) was utilized to assess empathy. The lack of ancillary neurological testing (involving neuroimaging or electroencephalography) meant that the underlying mechanisms could not be fully explored. Third, other tools that assess basic empathy-related functions (such as facial emotion recognition, emotion-related memory, and emotion-related decision making) should be used to investigate how cognitive empathy impairment occurs.

## 5. Conclusion

Collectively, our results to date indicated that chronic MSK pain just in outpatients could lead to social dysfunction, suggesting the importance to evaluate the empathetic function of this disease with suitable tools at an early age. These findings contribute to our understanding of the impaired empathy in chronic MSK pain patients and give doctors and physicians a starting point to consider the social and psychological factors in clinical decisions of chronic MSK pain, complementing current research and developing promising interventions.

## Figures and Tables

**Table 1:** Anatomical pain sites of the chronic musculoskeletal pain (MSK pain) patients.

Subject number	1	2	3	4	5	6	7	8	9	10	11	12	13	14	15	16	17	18	19	20	21	22
Shoulder									√	√			√	√	√	√	√					
Leg	√	√	√	√	√	√	√	√	√		√	√							√	√	√	√
Arm	√		√		√	√	√	√		√			√	√	√							√
Back	√	√			√				√		√	√			√	√	√	√	√			

“√”: site of chronic musculoskeletal pain.

**Table 2:** Demographic and psychological characteristics of chronic musculoskeletal pain (MSK pain) patients and healthy controls (HCs).

	MSK pain (*n* = 22)	HC (*n* = 26)	*t* (*χ*^2^)	*P*
Age	44.41 ± 7.94	40.08 ± 10.86	1.550	0.128
Education years	9.41 ± 4.08	11.64 ± 4.99	-1.650	0.102
Gender (male/female)	17/5	18/8	0.3903	0.532
Handedness (left/right)	0/22	1/25	1.2070	0.272
NRS	5.64 ± 2.81	0	NA	NA
SF-MPQ	7.36 ± 4.81	0.04 ± 0.200	NA	NA
Pain time (months)	22.21 ± 33.62	0	NA	NA
PSQI	6.91 ± 4.80	4.19 ± 2.30	2.550	0.014^∗^
Positive emotion	19.86 ± 6.94	21.69 ± 6.49	-0.950	0.351
Negative emotion	18.36 ± 7.14	15.19 ± 3.94	1.950	0.058
Depression	11.45 ± 3.46	10.04 ± 2.76	1.600	0.122
Anxiety	11.59 ± 3.75	10.04 ± 2.32	1.750	0.087
Stress	13.23 ± 3.57	12.62 ± 3.23	0.600	0.536

*P* represents level of significance from independent-samples *t*-test and chi-square as appropriate. NRS: Numerical Rating Scale; SF-MPQ: Short-Form McGill Pain Questionnaire; PSQI: Pittsburgh Sleep Quality Index. ^∗^*P* < 0.05; NA: not applicable.

**Table 3:** IRI fantasy (FS), perspective taking (PT), empathic concern (EC), and personal distress (PD) subscale scores in chronic musculoskeletal pain (MSK pain) and healthy control (HC) groups. The perspective-taking and fantasy subscales represent self-report cognitive empathy, while the empathic concern and personal distress subscales represent self-report emotional empathy.

		MSK pain (n = 22)	HC (n = 26)	*t*	*P*
Self-repot cognitive empathy	PT	21.86	21.27	0.442	0.660
	FS	18.14	17.77	0.282	0.779
Self-repot emotional empathy	EC	23.00	23.04	-0.039	0.969
	PD	19.50	19.58	-0.058	0.954

No significant difference between groups was found in IRI.

**Table 4:** Correlations of MET-C performance, including accuracy (mean accuracy and accuracy in positive/negative conditions) and task completion time, with positive/negative experienced emotion (PANAS) and other variables (age, education duration, PSQI, pain intensity, and pain duration).

	Age	Education years	PSQI	Positive PANAS	Negative PANAS	SF-MPQ	Pain time	Completion time	Mean accuracy^2^	Positive accuracy^2^	Negative accuracy^2^
Age	1										
Education years	-0.601^∗∗^	1									
PSQI	-0.032	0.536^∗^	1								
Positive PANAS	-0.031	-0.070	-0.095	1							
Negative PANAS	-0.233	0.134	0.224	0.390	1						
SF-MPQ	-0.109	0.089	0.275	-0.343	0.400	1					
Pain time	0.102	-0.017	0.186	-0.209	-0.044	0.090	1				
Completion time	0.497	-0.416	0.101	-0.002	0.024	0.184	-0.037	1			
Mean accuracy^2^	-0.237	0.286	0.036	0.341	-0.083	-0.606^∗∗^	-0.374	-0.436^∗^	1		
Positive accuracy^2^	-0.373	0.299	0.075	0.275	-0.074	-0.562^∗∗^	-0.331	-0.525^∗^	0.926^∗∗∗^	1	
Negative accuracy^2^	-0.031	0.205	-0.019	0.353	-0.060	-0.547^∗∗^	-0.362	-0.272	0.901^∗∗∗^	0.673^∗∗∗^	1

Values reported are Pearson correlation coefficients. PSQI: Pittsburgh Sleep Quality Index; PANAS: Positive and Negative Affectivity Scale; SF-MPQ: Short-Form McGill Pain Questionnaire. ^∗^*P* < 0.05, ^∗∗^*P* < 0.01, and ^∗∗∗^*P* < 0.001.

## Data Availability

The original data and related materials of this study can be accessed from the corresponding author upon request.
